# Pulmonary arteriovenous fistula in a rare location: The importance of excluding patent foramen ovale

**DOI:** 10.1016/j.jccase.2022.11.005

**Published:** 2022-12-07

**Authors:** Mitsutaka Nakashima, Takashi Miki, Yoichi Takaya, Rie Nakayama, Koji Nakagawa, Satoshi Akagi, Norihisa Toh, Teiji Akagi, Hiroshi Ito

**Affiliations:** Department of Cardiovascular Medicine, Okayama University Graduate School of Medicine, Dentistry, and Pharmaceutical Sciences, Okayama, Japan

**Keywords:** Pulmonary arteriovenous malformation, Paradoxical embolic stroke, Patent foramen ovale, Contrast saline transthoracic echocardiography

## Abstract

A 46-year-old woman with a history of repeated thromboembolic stroke and anti-phospholipid antibody syndrome was referred to our hospital. Saline contrast transthoracic echocardiography showed that microbubbles appeared in the left atrium within 4 heartbeats. Thus, she was initially suspected as having a patent foramen ovale with associated paradoxical embolism. However, no evidence of patent foramen ovale or atrial septal defect could be found using transesophageal echocardiography. Saline contrast transesophageal echocardiography showed that microbubbles flowed into the left atrium through the left superior pulmonary vein. Ultimately, she was diagnosed as having a pulmonary arteriovenous malformation located at the upper left pulmonary lobe using contrast computed tomography and pulmonary artery angiography. Pulmonary arteriovenous malformations are typically located in the lower lobe of either lung and, in bubble studies, contrast appears in the left atrium after 4 heartbeats. Here, the pulmonary arteriovenous malformation was in the upper lobe, and contrast appeared in the left atrium at an earlier time point: one associated with patent foramen ovale. These findings made it difficult to differentiate the two diseases initially. This case suggests that pulmonary arteriovenous malformation should be carefully considered, even if microbubbles appear in the left atrium early on a saline contrast transthoracic echocardiograph.

**Learning objective:**

Pulmonary arteriovenous malformation occasionally appears in the upper lobe. In these cases, microbubbles may appear in the left atrium after detection in the right atrium with a time-course that is suggestive of a patent foramen ovale. Therefore, diagnosis should be carefully confirmed by using other multimodal imaging tests, such as transesophageal echocardiography, contrast computed tomography, or pulmonary artery angiography.

## Introduction

Intracardiac or pulmonary shunts can cause right-to-left shunts. These in turn can cause paradoxical embolisms by allowing thrombi that originate in the venous vasculature into the systemic circulation [1], [2]. Patent foramen ovale (PFO) is the most common intracardiac shunt and is recognized as a risk of paradoxical embolism [2], [3], [4]. Pulmonary arteriovenous malformation (PAVM) is a rare disease in which abnormal direct vascular communication between the pulmonary artery and pulmonary vein occurs. PAVM can also cause paradoxical embolism as with PFO [2]. In general, the differential diagnosis of PFO or PAVM is made possible by observing the timing with which microbubbles appear in the left atrium on contrast saline transthoracic echocardiography (TTE) [5], [6], [7]. Here, we describe a case of non-hereditary, isolated PAVM with a history of repeated paradoxical embolism. The initial diagnosis was complicated by the fact that microbubbles appeared in left atrium with a time-course that was suggestive of PFO.

## Case report

**Fig. 1:**
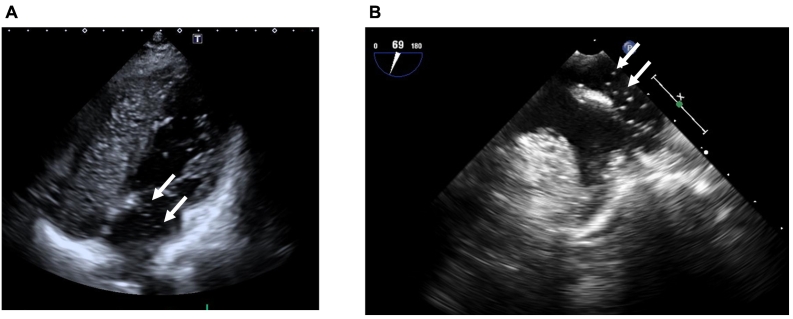
Saline contrast tests. (A) Microbubbles in the left atrium three cardiac cycles after detection in the right atrium on transthoracic echocardiography (arrows). (B) Microbubbles flowing into the left atrium through left upper pulmonary vein on transesophageal echocardiography (arrows).

**Fig. 2:**
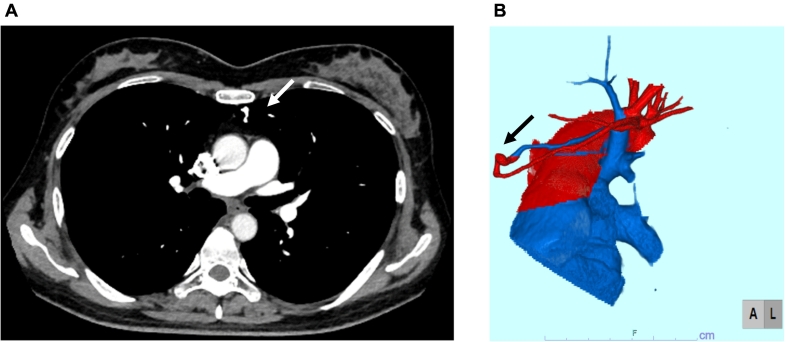
An abnormal vessel (2.2 mm in diameter) in the left pulmonary upper lobe on contrast computed tomography (arrows). (A) axial slice, (B) three-dimensional reconstruction of the pulmonary artery and vein.

**Fig. 3:**
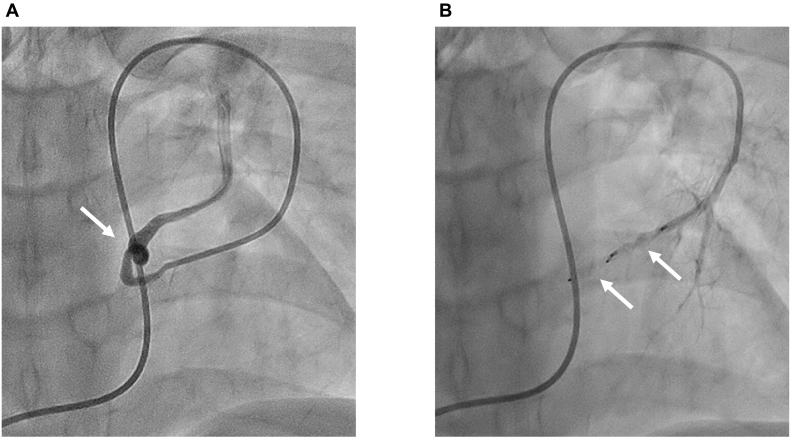
Selective pulmonary artery angiography on left pulmonary artery A3. (A) Pulmonary arteriovenous malformation draining into left upper pulmonary vein (arrow). (B) Completely interrupted blood flow into pulmonary arteriovenous malformation after implantation of 4 mm and 5 mm of Amplatzer Vascular Plug 4 (Abbott, Minneapolis, MN, USA) (arrows).

A 46-year-old woman was referred to our hospital because of history of repeated stroke twice in two years. Her stroke was diagnosed as thromboembolic stroke by neurologists. She also presented with the comorbidity of anti-phospholipid antibody syndrome. Cyanosis was not observed, either at rest or during exercise. She did not have any symptoms of vasculitis such as fever or exanthema. She had situs solitus. Thus, she was receiving anticoagulant therapy by warfarin at the dosage of 2.25 mg daily. She had no relevant family medical history. Atrial fibrillation had not been detected by Holter monitoring. Her peripheral oxygen saturation was 98 %. On saline contrast TTE, both at rest and during Valsalva maneuver, microbubbles appeared in the left atrium as grade 3 within three cardiac cycles after detection in the right atrium (Fig. 1A) [7]. There were no significant valvular abnormal finding including Libman-Sacks endocarditis. However, intracardiac shunt flow was not detected by color Doppler. Nevertheless, she was initially suspected as having a PFO that led to paradoxical embolism. However, there was no evidence of PFO or atrial septal defect on transesophageal echocardiography (TEE), in dispute with the earlier observation of saline contrast TEE. On further investigation, the microbubbles were observed flowing into the left atrium through the left superior pulmonary vein rather than through a PFO (Fig. 1B). Considering these findings, a right-to-left shunt through the pulmonary vein to the systemic circulation was suspected. Contrast computed tomography showed an abnormal vessel that was 2.2 mm in diameter and originated from the left upper pulmonary artery and drained into the left upper pulmonary vein (Fig. 2). Other arteriovenous malformation including cerebral or abdominal was not detected. There was no discernable deep venous thrombosis or pulmonary embolism on contrast computed tomography. On carrying out selective pulmonary artery angiography on the left pulmonary artery A3, a PAVM that drained into the upper left pulmonary vein was detected (Fig. 3A). Thus, this case was finally diagnosed as non-hereditary, isolated PAVM. Percutaneous transcatheter occlusion was performed. After implantation of 4 mm and 5 mm of Amplatzer Vascular Plug 4 (Abbott, Minneapolis, MN, USA), the blood flow into the PAVM was completely interrupted (Fig. 3B). Sixteen months after percutaneous transcatheter occlusion (when this case report was submitted), thromboembolic stroke had not recurred.

## Discussion

We describe here a rare case of PAVM with a history of repeated thromboembolic stroke. Initial diagnosis by contrast saline TTE was difficult because microbubbles appeared in the left atrium within three cardiac cycles after detection in the right atrium, indicative of a PFO. Saline contrast TEE indicated the existence of a right-to-left shunt with its outflow in left superior pulmonary vein. She was finally diagnosed as having PAVM by contrast computed tomography and pulmonary artery angiography.

PAVM is a disease in which a shunt between the pulmonary artery and pulmonary vein is present [8]. PAVM causes various symptoms, such as cyanosis, brain abscesses, and paradoxical embolism [2]. Usually, PAVM appears in lower lobes due to the increased pulmonary blood flow at the base of the lung. In some cases, multiple shunts are present [9]. Hereditary hemorrhagic telangiectasia is frequently associated with PAVM [8]. This case suffered paradoxical embolism caused by PAVM with a single shunt, and she did not display any specific clinical signs of hereditary hemorrhagic telangiectasia, such as epistaxis or family history. She was therefore diagnosed as having a non-hereditary, isolated case of PAVM. Although the incidence of symptoms is reported to be greater in patients with multiple shunts [8], this patient had a history of repeated paradoxical embolism associated with isolated PAVM. The comorbidity of anti-phospholipid antibody syndrome with PAVM might increase risk of paradoxical embolism.

The gold standard for diagnosis of PAVM is computed tomography pulmonary angiography [9]. Three-dimensional reconstruction of the pulmonary artery and pulmonary vein by computed tomography is useful, not only for the diagnosis of PAVM but also in carrying out an anatomical assessment for the purpose of treatment [10]. Saline contrast TTE or TEE is also recommended for risk assessment in PAVM patients [5], [9].

To discriminate between PAVM and PFO, the difference in the timing of the appearance of microbubbles in the left atrium is typically used [5], [6], [7]. Our findings show that, in cases where microbubbles appear in the left atrium on saline contrast TTE or TEE with a time-course that implies the existence of a right-to-left shunt, it is necessary to take steps to distinguish PFO from PAVM. Typically, in cases with PFO, microbubbles appear in the left atrium within three cardiac cycles after either the release of the Valsalva maneuver or the appearance of contrast solution in the right atrium. In contrast, in PAVM, microbubbles would appear after four cardiac cycles. However, in the present case, microbubbles appeared in the left atrium within three cardiac cycles after detection in the right atrium. This led us to an erroneous initial diagnosis of PFO. PAVM has been reported as most commonly appearing in the lower lobes of the lung: those which are supplied by vessels that arise from a relatively distal point on the pulmonary artery [9]. In this case, the PAVM appeared in the left upper lobe, which is supplied by vessels that arise from a relatively proximal point on the pulmonary artery. This atypical appearance site might explain the early appearance of microbubbles on saline contrast test because the distance between the heart and the PAVM in this case was shorter than in typical cases. TEE was an efficient means of confirming that there was no right-to-left shunt at the level of the atrium and that microbubbles flowed into the left atrium through left superior pulmonary vein. In summary, this case suggests that PAVM should be carefully considered as an alternative to a PFO, even in cases where microbubbles appear in the left atrium within three cardiac cycles after the release of the Valsalva maneuver or the appearance of contrast solution in the right atrium on saline contrast TTE or TEE.

In respect to treatment strategies for paradoxical embolism, there are the different indications between PAVM or PFO. In cases with PFO, PFO closing should be considered based on risk assessment for paradoxical embolism such as anatomical features [4]. On the other hand, the occlusion procedure for PAVM is reported to be reasonable, even if PFO is coincident [2]. Thus, it seems to be important to detect PAVM for cases with history of paradoxical embolism, even if PFO is suspected as its cause.

In conclusion, we present a rare case of isolated PAVM that presented difficulties in differential diagnosis. In cases with a history of embolic stroke of unknown cause, PAVM should be considered as a diagnosis, even if saline contrast TTE or TEE are suggestive of PFO.

## Declaration of competing interest

The authors declare that there is no conflict of interest.
